# Elevational constraints and climatic buffering: comparison of Pierinae and Coliadinae across Taiwan Island and mainland China

**DOI:** 10.1186/s12983-026-00621-6

**Published:** 2026-06-22

**Authors:** Riaz Hussain, Lianxi Xing, Yuan Hua

**Affiliations:** 1 College of Life Sciences, Northwest University, Xi’an, 710069 China; 2 Shaanxi Key Laboratory for Animal Conservation (Northwest University), Xi’an, 710069 China; 3 Key Laboratory of Resource Biology and Biotechnology in Western China, Northwest University, Ministry of Education, Xi’an, 710069 China

**Keywords:** Pierinae, Coliadinae, Distribution pattern, Mainland–island comparison, MaxEnt model, Climate change

## Abstract

Different elevational gradients and anthropogenic pressures between island and mainland systems mediate discrete climatic sensitivities and distribution trends in butterfly lineages. We assess the comparative distributional changes of Pieridae (Pierinae and Coliadinae) on Taiwan Island and mainland China. Across Taiwan Island, the geographic range of Pieridae is mainly limited to lowlands due to the sharp decline in temperature with rising elevation. In mainland China, both reduced temperature and low precipitation at upper elevations shape Pieridae’s spatial pattern. Across Taiwan, both subfamilies showed positive associations with human disturbances at greater elevations, while Pierinae showed a positive relationship with thick vegetation cover at lower elevations. Throughout mainland China, Pieridae exhibited negative associations with human disturbances and vegetation density at higher elevations. According to the MaxEnt results, across Taiwan Island, elevation is mainly responsible (80.6%) for the distribution of Pierinae, while maximum temperature of warmest month (Bio5) is mainly influencing (53.7%) the distribution of Coliadinae. Initially Pierinae exhibited an increase in highly suitable regions across the three historical periods, followed by a decrease. Conversely, Coliadinae showed an initial contraction, followed by an expansion. Future global warming may result in a reduction of highly appropriate habitats for both subfamilies without significant evidence of upslope migration. Most Pieridae species are projected to undergo habitat contraction, resulting in decreased species diversity. These outcomes indicate stronger ecological constraints and higher vulnerability to Pieridae diversity in island systems, highlighting the necessity of implementing regional conservation strategies.

**Supplementary Information:**

The online version contains supplementary material available at https://doi.org/10.1186/s12983-026-00621-6.

## Introduction

Global warming is reshaping ecosystems at an unprecedented rate, driving shifts in biodiversity through changes in species distributions, behavior, and biotic interactions. Species unable to track suitable conditions may face an elevated risk of extinction [1–3], particularly regional and endemic taxa [3–6]. Insects, specifically butterflies, are widely recognized as excellent indicators of climate change due to their short generation period, great mobility, and specialized habitat needs. Increasing evidence shows that butterfly distributions are shifting in response to changes in temperature, precipitation, and habitat conditions [1–4, 7]. In mainland China, global warming has been associated with declines in butterfly diversity, contraction of appropriate regions, and pronounced upslope range shifts [1, 2, 7]. In contrast, island ecosystems often exhibit distinct responses to climate change, where species often show latitudinal shifts rather than elevational ones, with biodiversity losses concentrated at lower elevations where habitat degradation and human population density are greatest [6, 8, 9]. By comparison, mainland regions more commonly facilitate elevational adjustments, which can partially mitigate climate-driven biodiversity loss [1, 2, 7, 8]. These contrasting response patterns indicate that geographic setting plays a critical role in shaping how species track climate change.

The mechanisms underlying these differences are rooted in fundamental biogeographic and landscape characteristics. Island biodiversity is primarily governed by area, habitat isolation, stochastic processes, and source-sink dynamics, which regulate colonization and extinction rates and often result in simpler community structures [5, 6, 8, 9]. Additionally, the steep topography and constrained lowland areas of islands—particularly Taiwan—can [10–12] limit species dispersal and spatial flexibility, increasing vulnerability to environmental change and anthropogenic pressures [5, 6, 8, 9]. Such constraints reduce the availability of alternative habitats and restrict movement pathways under changing climates. In contrast, mainland systems offer greater habitat continuity, broader environmental gradients, and larger species pools, thereby enhancing dispersal capacity and allowing species to track environmental change across broader geographic and elevational ranges [1, 2, 7, 8]. These differences are especially pronounced for butterflies, whose distributions are strongly influenced by dispersal capacity, habitat heterogeneity, and resource availability, making them highly sensitive to landscape fragmentation [1, 2, 5–7, 9]. Despite the strong theoretical framework of island biogeography and well-established differences between mainland and island systems, comparative studies of butterfly distributions across mainland China and Taiwan Island remain limited. To address this gap, investigating Pierinae and Coliadinae across these contrasting regions offers a critical opportunity to clarify how habitat isolation, topographic constraints, and environmental gradients jointly influence species’ climatic sensitivity and distributional dynamics under past and ongoing climate changes.

The Maximum Entropy (MaxEnt) model [13] is widely used in ecological and biogeographic research for forecasting species distribution and informing biodiversity protection [1, 7] and control [14] across different periods. During the Last Glacial Maximum (LGM), lower temperatures facilitated cold-adapted species to expand into newly suitable regions, whereas species preferring warmer conditions receded to refugia [1, 15]. Following the LGM, post-glacial dispersal reshaped species distributions, structuring present-day patterns [1, 15]. Understanding how biodiversity responded to past climate alterations is critical for predicting future responses to ongoing global warming, providing a robust framework for identifying climatic references among species [1, 2, 15].

Building on this context, understanding large-scale biodiversity patterns and the processes underlying species responses is essential for effective conservation planning [1, 2, 7]. China, with its complex topography and extensive mountain systems, is home to over 2,000 butterfly species [16, 17]. Pierinae and Coliadinae subfamilies are widely distributed across mainland China, from near sea level to 6,000 m. In mainland China, both subfamilies expanded their highly suitable habitats from the Last Interglacial (LIG) to LGM, but most species are projected to experience future habitat contraction and upslope range shifts, with consequent declines in species richness [1]. Across Taiwan Island, comprehensive assessments of Pieridae distributions across historical and future periods remain lacking. Furthermore, researchers have not thoroughly examined the underlying determinants controlling the distribution trends of both subfamilies across elevational gradients throughout Taiwan Island and mainland China, nor have they systematically compared these patterns between the two regions. This knowledge gap limits effective conservation planning and poses potential risks to Pieridae diversity, as it hinders the development of targeted strategies to mitigate threats to these butterfly populations. Hence, we compiled detailed occurrence data for both Pierinae and Coliadinae to (1) identify the spatial density and distribution patterns of both subfamilies and investigate how environmental factors shape their distribution trends; and (2) analyze the changes in distribution on Taiwan Island throughout the LIG, LGM, 1970–2000 (Current), and future projections, and compare these patterns with those observed in mainland China.

## Materials and methods

### Study site

**Fig. 1:**
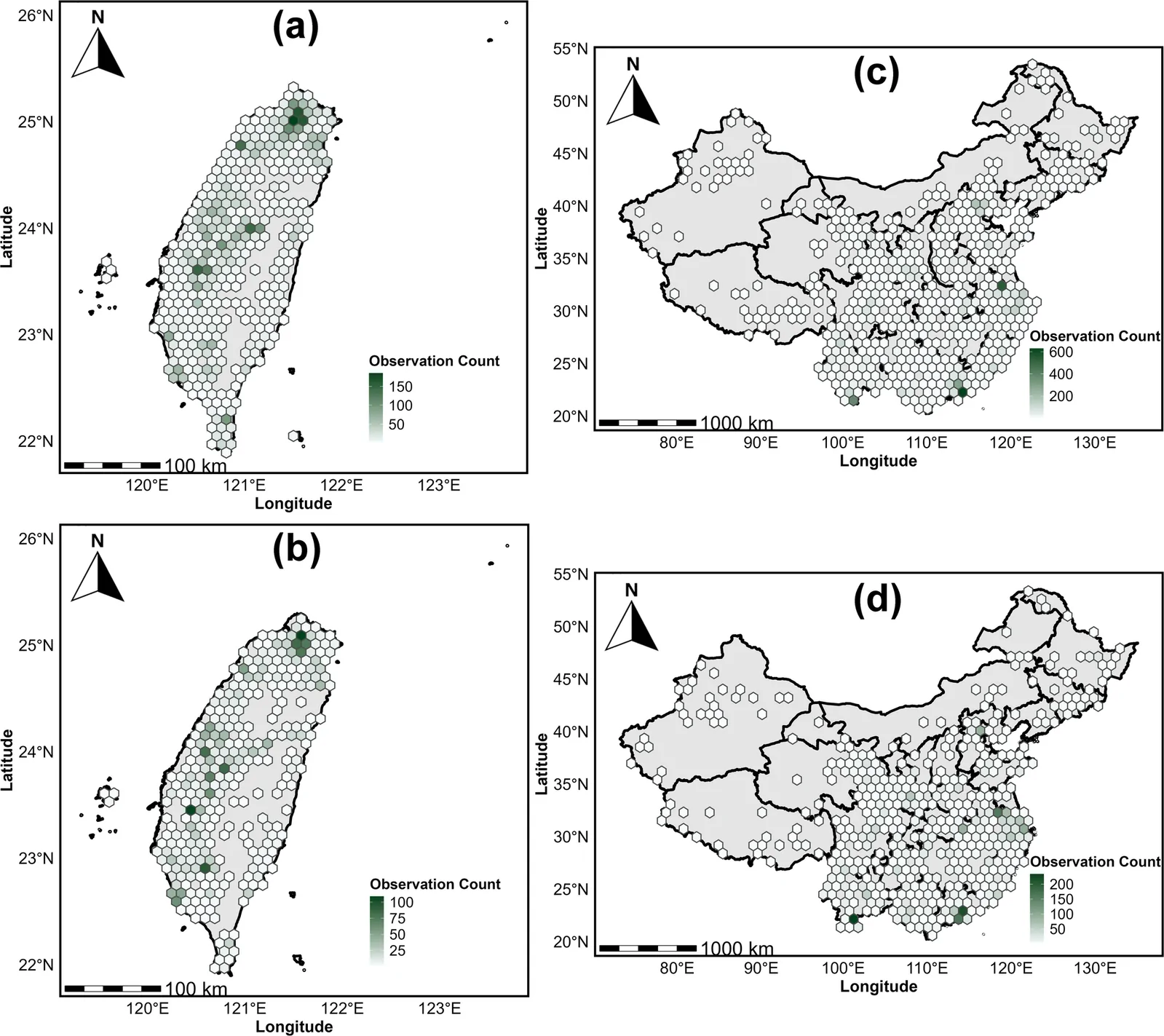
Hexbin density map of Pieridae across Taiwan Island and mainland China: (**a**) Pierinae and (**b**) Coliadinae observations on Taiwan Island; (**c**) Pierinae and (**d**) Coliadinae records in mainland China

This study consists of Taiwan Island and mainland China (Fig. 1). Taiwan Island is located in East Asia, positioned between the East and South China Seas in the northwestern Pacific Ocean [18, 19], with mainland China to the northwest, Japan to the northeast, and the Philippines to the south. It is located around 180 km away from the southeastern coast of mainland China and encompasses a total area of 36,193 square kilometers. The island features several peaks exceeding 3,500 m. The eastern mountainous regions are densely forested and host a diverse array of wildlife, whereas land use in the western and northern lowlands is characterized by intensive development [10–12]. The northern and central areas of Taiwan Island are subtropical, whereas the southern part is tropical, and the mountainous zones are temperate. The island receives an annual average rainfall of 2,600 millimeters (mm). The whole island endures hot and humid conditions from June to September. Typhoons predominantly occur from July to September. From November to March (winter), the northeast part of the island undergoes consistent rainfall, but the central and southern regions predominantly enjoy sunny weather [20].

Mainland China, as part of East Asia, has diverse geography, featuring the Himalayan mountain range in the southwest and extensive plains in the east. The altitude varies considerably, from sea level on the eastern coast to over 8,844 m on the Tibetan Plateau. Mainland China exhibits a range of climatic patterns, from temperate regions in the north to subtropical weather in the south [16]. The northern side of mainland China exhibits two prolonged seasons (winter and summer), interspersed with short transitional periods. Winters are characterized by low humidity and cold temperatures, often dropping below the freezing point. Summers exhibit elevated temperatures accompanied by significant levels of precipitation. The southern part of mainland China exhibits perennial greenery; therefore, elevated temperatures, high precipitation, and increased humidity of the area foster optimal conditions for biodiversity. Winters are brief, occurring from January to March, but can be cold [21, 22].

### Occurrence data

The occurrence data of Pieridae (Pierinae and Coliadinae) for Taiwan Island were downloaded from the Global Biodiversity Information Facility (GBIF, https://www.gbif.org/) and iNaturalist (https://www.inaturalist.org/) using the ‘rgbif’ [23] and ‘rinat’ [24] packages in R v4.4.3 [25], respectively. The occurrence data were also retrieved from the China National Knowledge Infrastructure (CNKI, https://www.cnki.net/). These databases were accessed on January 13, 2025. Missing coordinates were located using Google Earth (https://earth.google.com/), following Hussain et al. 2026 and Haroon et al. 2026 [14, 26]. In total, 9,683 occurrence records were noted for 33 Pieridae species, including 6,592 for Pierinae and 3,091 for Coliadinae. Among the total species gathered, 23 were classified as Pierinae and 10 as Coliadinae (Fig. S1). Furthermore, for the analysis of mainland China, the Pierinae and Coliadinae distribution data used by Hussain et al. 2025 (our previous study) [1] were applied in the current study.

To mitigate spatial autocorrelation in occurrence data, we refined the dataset by retaining a single point within each 1,000 m, 5,000 m, and 10,000 m radius, employing a geographic distance-based approach using the ‘lwgeom’ and ‘sf’ packages in R v4.4.3 [27, 28]. The output from the 1,000 m radius yielded the most reliable model performance, which was therefore utilized in this study. Finally, a total of 1,875 Pierinae and 1,026 Coliadinae distribution points from Taiwan Island, along with 3,434 Pierinae and 1,927 Coliadinae distribution points from mainland China, were utilized in subsequent analysis. For Pierinae and Coliadinae, the hexbin density maps for Taiwan Island (size 10 km per grid) and mainland China (size 100 km per grid) and the kernel density curves were drawn using the ‘ggplot2’ [29] and ‘sf’ [30] packages in R v4.4.3 [28]. Different grid sizes were used for both regions in accordance with the regional geographical extent.

### Environmental variables

Environmental variables, including climate, land characteristics, and anthropogenic activities, influence the survival rate, reproduction, larval development, habitat availability, and dispersal of butterflies [2, 31]. This study chose 6 types of environmental datasets, which included 26 environmental variables: 19 bioclimatic, 2 topographic (elevation and aspect), 2 anthropogenic (human population and urbanization), 1 soil (soil moisture), 1 vegetation (normalized difference vegetation index), and 1 land surface variable (land surface temperature) (Table S1). Overall, 19 bioclimatic factors were obtained from the WorldClim database (http://www.worldclim.org/). The WorldClim 1.4 dataset [32] was chosen for paleoclimatic data (Last Glacial Maximum (LGM)). We picked CCSM4 (the Community Climate System Model version 4) [33] for its excellent predictive performance and efficacy in forecasting the effects of climate change on the distribution of flora and fauna [34, 35].

The WorldClim 2.1 dataset [1, 36] was chosen for Current (1970–2000) and future (2021–2040, 2081–2100) climate data. The data were derived from the Coupled Model Intercomparison Project Phase 6 (CMIP6). The global climate model (GCM)—ACCESS-CM2—was selected. We projected the suitable regions based on future environmental factors under a stringent mitigation scenario (SSP1–2.6) and elevated greenhouse gas emissions (SSP5–8.5) [37]. Between the two topographic factors, the elevation factor was downloaded from the WorldClim database. The aspect values were calculated from the elevation factor using the ‘terrain’ function of the raster package in R v4.4.3 [26, 28, 38]. The rest of the factors were obtained from the Google Earth Engine (GEE, https://developers.google.com/earth-engine/). The WorldClim and GEE databases were assessed on June 5, 2025.

We only used 19 bioclimatic and 2 topographic variables in the MaxEnt model (Table S1). With the exception of bioclimatic variables, we assumed that topographic factors stayed constant across various periods because their changes lag far behind climate change and the absence of data [39]. Each environmental factor was clipped to the boundaries of the study area using the R package ‘terra’ [40], resampled to a consistent spatial resolution [39] of 2.5′ (\~4.3 km), and transformed to ASC format.

### Evaluation of species occurrence points

Using the ‘letsR’ package [41], a presence-absence matrix (PAM) was created at a resolution of 2.5′ (\~4.3 km) by overlaying species coordinates data with a uniform grid across Taiwan Island and mainland China. The PAM underwent additional processing with EstimateS v9.1 [42] to draw species accumulation curves, calculating bootstrap mean and observed species richness (S.est) with respect to sampling effort (grid cells) [1, 43].

### Environmental variables selection and model calibration

To mitigate the chance of excessive fit in the MaxEnt model arising from robust connections between the 21 factors (Table S2) generally for both subfamilies (Pierinae and Coliadinae) (Figs. S2–S3)) and particularly for every species, the correlation among all variables was thoroughly analyzed using the ‘corrplot’ package [44]. The contribution rates of all variables used in the MaxEnt model were computed for both subfamilies (Table S2) and every species individually. The decision to select criteria for the final MaxEnt model included maintaining the six most significant variables, generally for both subfamilies (Figs. S2–S4) ; Table S2] and specifically for each species exhibiting significant contribution values between pairs of highly correlated factors (|*r*| ≥ 0.8) [1, 14, 26].

‘ENMeval’ [45] was applied in R v4.4.3 to mitigate model overfitting generally for Pierinae and Coliadinae and specifically for each species (Table S3) by adjusting parameters of feature combinations [linear (L), quadratic (Q), product (P), threshold (T), and hinge (H)) and regularization multiplier (RM) value (1–3) with increments of 1, combined with the feature types (L, LQ, H, LQH, LQHP, and LQHPT), and calculating the Akaike information criterion (AICc) values using ‘block’ [1, 14, 26]. LQHP and RM value 2 represent the model with the minimal delta AICc for Pierinae, but for Coliadinae, LQHPT and RM value 2 denote the model with the lowest delta AICc. The number of distribution points and settings used to construct the model for each species is given in Table S3. Based on the fewer distribution points and poor MaxEnt results, ten species were skipped from the simulation analysis. Model outputs with the minimal delta AICc for the remaining 23 species were calculated (Table S3). A 75% random sample was utilized for model construction, with the remaining 25% reserved for model evaluation [1, 14].

Background sampling in the MaxEnt model was initially performed with the default method generally for Pierinae and Coliadinae and specifically for each species, which selects 10,000 random locations throughout the research area [1, 14, 26]. To mitigate potential sampling bias and improve model efficacy, we additionally evaluated two alternative methodologies: (i) generating 10,000 background points through kernel density estimation of occurrence records and (ii) integrating a bias file obtained from kernel density estimation to inform background selection [46, 47]. Among these, the kernel density-based background point method yielded the most reliable and ecologically valid predictions, leading to its adoption in the final models.

The paleoclimatic variables were linearly rescaled to align with the value ranges of Current (1970–2000) variables, using the ‘raster’ package in R v4.4.3 [1, 28, 38]. This maintained ecological gradients while reducing bias from absolute value discrepancies. Suitable areas were classified into the following four levels: negligible-suitable areas (0–0.2), low-suitable areas (0.2–0.40), medium-suitable areas (0.40–0.60), and high-suitable areas (0.60–1.00) [1, 14]. The area corresponding to each suitable class was calculated in square kilometers (km^2^) [2]. Areas under the receiver operating characteristic (ROC) curves were generated with area under the curve (AUC) values averaged with ten replicated runs and utilized for model evaluation along with the true skill statistic (TSS) [1, 14, 48].

We utilized a species-level, stacked species distribution modeling (S-SDM) approach [1] to examine the distributions and species richness of 23 Pieridae butterflies. The corresponding six most significant, uncorrelated (|*r*| < 0.8) environmental parameters were selected as predictors for each species (Table S3) [1, 14]. Spatial thinning was employed to reduce sample bias by removing multiple points inside identical raster cells. S-SDM was developed in R v4.4.3 [28], employing MaxEnt v3.4.4 [13] through the ‘dismo’ package [49]. For every species, we kept the settings for assessment of the model efficiency, feature class, regularization multiplier, training, testing, and background points the same as mentioned above (see Environmental variables selection and model calibration). The threshold that optimized TSS was utilized to convert continuous predictions into binary presence-absence maps. The species-specific binary maps were aggregated to create a richness map. The area associated with every suitability class for each species was calculated in km² [1, 2]. To enable comparison utilizing probability-style classes (0–1 range), the richness score in every cell was standardized. The standardized richness proportions were categorized into four levels (see Environmental variables selection and model calibration) [1, 2, 14] to illustrate relative species richness spatially.

### Elevational distribution analysis

To clarify the distributional differences encountered by Pierinae and Coliadinae across Taiwan Island and compare them with their distribution in mainland China, we divided the occurrence data of Pierinae and Coliadinae at the median elevation (‘low’ and ‘high’ elevation groups). This method offers a resilient, data-driven threshold that exhibits less sensitivity to uneven distributions or extremes. To find the determinants controlling the distribution of Pierinae and Coliadinae across the elevational gradient, the occurrence points of both subfamilies and regions were used separately. Data of each variable (Table S1) were extracted for every occurrence point from their corresponding raster layers using the ‘terra’ package in R v4.4.3 [28]. We first conducted the correlation analysis for Pierinae and Coliadinae to determine the most strongly associated variables with elevation, utilizing Pearson’s correlation coefficient [44]. The absolute strength of variables was ranked based on the magnitude of their correlation. The association of the top eight variables with elevation for both subfamilies was visualized using the ‘ggplot2’ package [29].

Subsequently, for both subfamilies, we constructed a linear mixed-effects model (LMM) utilizing the ‘lmer’ function from the ‘lme4’ package [50] to analyze their distribution against the elevation gradient in relation to the environmental variables. The environmental variables were scaled through mean centering and standard deviation division to facilitate model convergence and enable comparison of effect sizes. The assessment of model fit was conducted using marginal *R*², which represents the variance explained by fixed effects, using the ‘MuMIn’ package in R v4.4.3 [28, 51].

### Forecasting elevational changes

To statistically validate the potential shift of Pierinae and Coliadinae across the elevation gradient on Taiwan Island, we performed an elevation-based analysis. We used the MaxEnt model algorithm in R 4.3.2, utilizing a 50th percentile threshold [7, 52], to assess potential elevational shifts in both subfamilies caused by climate change, applying the same variables (see Environmental variables) and settings (see Environmental variables selection and model calibration) as previously mentioned. Both subfamilies are widely spread throughout the study area; therefore, we utilized the 50th percentile threshold [7, 52] to delineate acceptable ecological regions. To statistically evaluate differences in mean elevation, a Welch two-sample *t*-test was conducted [7, 53]. Finally, kernel density plots were generated to visualize a potential shift in elevation using the ‘ggplot2’ package [29].

## Results

### Distribution patterns

Despite the extensive documentation and abundant geographical data on Pieridae (Pierinae and Coliadinae), insufficient collection persists as a main issue in biogeographic studies. In the current research, the species accumulation curves for Pierinae (Fig. S5(a)) and Coliadinae (Fig. S5(b)) across Taiwan Island and mainland China (Fig. S5(c–d)) reached clear asymptotes. These outputs demonstrated a high level of sampling completeness, and the occurrence data we downloaded from different databases were enough for subsequent analyses.

Across Taiwan Island, the hexbin density map of Pierinae (Fig. 1(a)) showed a notable concentration of occurrences in Taipei City, whereas Coliadinae (Fig. 1(b)) revealed a significant aggregation of occurrences in Taipei and Chiayi Cities. Throughout mainland China, Pierinae showed high abundance in Hong Kong (Fig. 1(c)), while Coliadinae are concentrated in Guangzhou City and Xishuangbanna Dai Autonomous Prefecture (Fig. 1(d)).

**Fig. 2:**
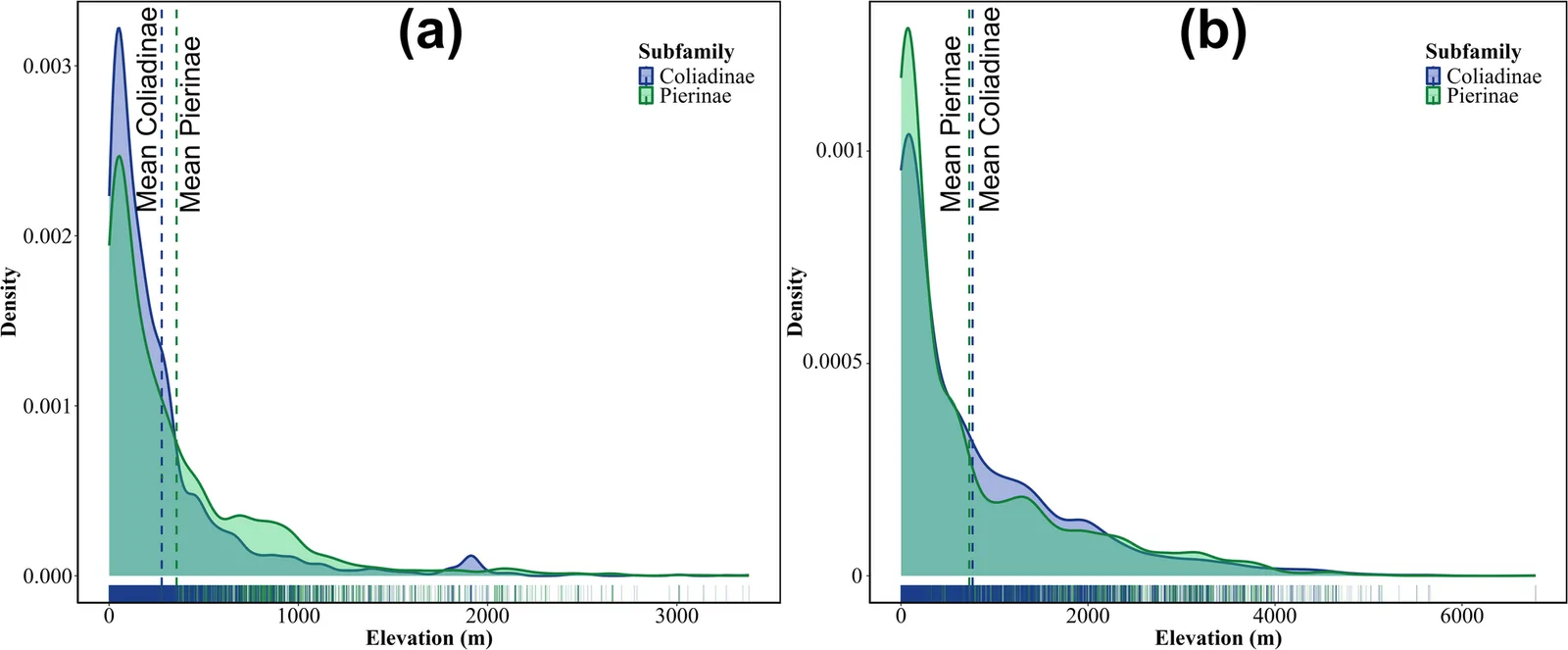
Elevational distribution of the subfamilies Pierinae and Coliadinae throughout (**a**) Taiwan Island and (**b**) mainland China. Kernel density curves demonstrate the frequency distribution of occurrence records throughout the elevation gradient for each subfamily, with green denoting Pierinae and blue indicating Coliadinae. Dashed vertical lines show the mean elevation of occurrence for each subfamily. Rug lines at the base of the plot indicate individual occurrence points, offering a visual representation of data density along the gradient

Elevation significantly influenced the distribution of Pierinae and Coliadinae on Taiwan Island and mainland China. Throughout Taiwan Island, in the case of Pierinae, 25 occurrence points were noticed at elevations above 2,500 m, while only 7 occurrence points were reported for Coliadinae. In contrast, in mainland China, at elevations above 2,500 m, 573 occurrence points were noted for Pierinae and 251 for Coliadinae. The kernel density curves indicate that across Taiwan Island, Pierinae inhabit comparatively higher mean elevations than Coliadinae. The mean elevation of Pierinae and Coliadinae across Taiwan Island was 429 m and 333 m, respectively (Fig. 2(a)). In contrast, in mainland China, Coliadinae reach their maximum at a slightly higher mean elevation than Pierinae. The mean elevation of Pierinae and Coliadinae was 819 m and 897 m, respectively (Fig. 2(b)). Notably, across Taiwan Island, only a limited number of species were recorded above 3,000 m. Pierinae species found over 3,000 m were *Aporia agathon*, *Delias pasithoe*, and *Prioneris thestylis*. Coliadinae species found beyond 3,000 m were *Catopsilia pomona*, *C. pyranthe*, *Eurema hecabe*, and *E. mandarina*. In mainland China, 56 Pierinae and 34 Coliadinae species were recorded above 3,000 m.

### Environmental drivers across the elevation gradient

In both Taiwan Island and mainland China, the correlation analysis for both subfamilies and their distribution along the elevation gradient demonstrated significant differences. In the case of Taiwan Island, the eight primary environmental factors significantly negatively affecting the distribution of Pierinae (Fig. S6(a)) and Coliadinae (Fig. S6(b)) along the elevational gradient were exclusively temperature-related, signifying a pronounced decrease in temperature with rising elevations. These changes led to a decrease in Pierinae and Coliadinae diversity at higher elevations. In the case of mainland China, the top eight environmental variables controlling the distribution of Pierinae (Fig. S7(a)) and Coliadinae (Fig. S7(b)) along the elevation gradient were temperature- and precipitation-related. These variables showed significant negative correlations with elevations, which in turn reduces the distribution of both subfamilies along the elevational gradient. In contrast, mean diurnal range (Bio_2) showed a positive correlation with elevations, supporting an increase in the distribution of both subfamilies along the elevation gradient.

The comparison of areas situated either below or above the median elevations for Pierinae (429 m) and Coliadinae (333 m) on Taiwan Island (Fig. 2(a)) revealed significant environmental disparities. Low-elevation locations exhibited markedly higher temperatures across all temperature parameters (Figs. S8–S9). Furthermore, the comparison of areas situated either below or above the median elevations for Pierinae (819 m) and Coliadinae (897 m) in mainland China (Fig. 2(b)) showed substantial environmental differences. Low-elevation locations exhibited markedly higher temperature and precipitation across all factors (Figs. S10–S11). These results indicate that temperature is mainly responsible for the significant decline of Pierinae and Coliadinae occurrences at higher elevations across Taiwan Island, while in the case of mainland China, both temperature and precipitation are controlling their distribution across the elevation gradient.

The linear mixed-effects model (LMM) showed a complex interaction of environmental variables influencing the distribution of Pierinae (marginal *R*² = 0.824) and Coliadinae (marginal *R*² = 0.8496) across the elevation gradient throughout Taiwan Island. In the case of Pierinae (Table S4), variables such as mean temperature of driest quarter (Bio_9), mean temperature of warmest quarter (Bio_10), and precipitation of wettest quarter (Bio_16) were extremely positively linked with occurrences at higher elevations. Moderate positive correlations were noted with temperature (Bio_2), mean temperature of wettest quarter (Bio_8), precipitation of driest month (Bio_14), and urbanization (Urb). Conversely, the greatest significant negative effects were noted for annual mean temperature (Bio_1), annual precipitation (Bio_12), precipitation of wettest month (Bio_13), land surface temperature (LST), and normalized difference vegetation index (NDVI). In the case of Coliadinae (Table S4), temperature (Bio_8) was highly positively correlated with occurrences at higher elevations. Mild positive relationships were seen with precipitation (Bio_16) and human population (HP). In contrast, precipitation (Bio_13) and LST showed significant negative correlations with elevations. The findings indicate that temperature and precipitation are responsible for controlling the distribution of Pieridae across the elevation gradient. Additionally, moderate anthropogenic activities are responsible for the occurrences of both subfamilies at higher elevations, while vegetation cover is responsible for Pierinae distribution at lower elevations.

Furthermore, the LMM demonstrated a complex interaction of climatic variables affecting the distribution of Pierinae (marginal *R*² = 0.948) and Coliadinae (marginal *R*² = 0.89) along the elevation range in mainland China. Throughout the elevation gradient, the distribution of Pierinae and Coliadinae (Table S5) displayed significant positive associations with temperature (Bio_1 and Bio_2) and precipitation of driest quarter (Bio_17). The subfamily Pierinae also showed a strong positive correlation with precipitation of warmest quarter (Bio_18). Conversely, both Pierinae and Coliadinae exhibited significant negative associations with temperature (isothermality (Bio_3), temperature seasonality (Bio_4), Bio_8, and temperature of coldest quarter (Bio_11)), precipitation of coldest quarter (Bio_19), urbanization (Urb), and NDVI. Pierinae also showed strong negative correlations with precipitation (Bio_12 and Bio_16), whereas Coliadinae showed strong negative correlations with SM. These results suggest that both temperature and precipitation are responsible for Pieridae occurrences across the elevation gradient. Furthermore, increased anthropogenic activities and vegetation cover are responsible for their decreased occurrences at higher elevations. These results suggest that temperature, precipitation, human activities, and habitat conditions significantly influence Pieridae populations.

## Assessment of MaxEnt model

**Fig. 3:**
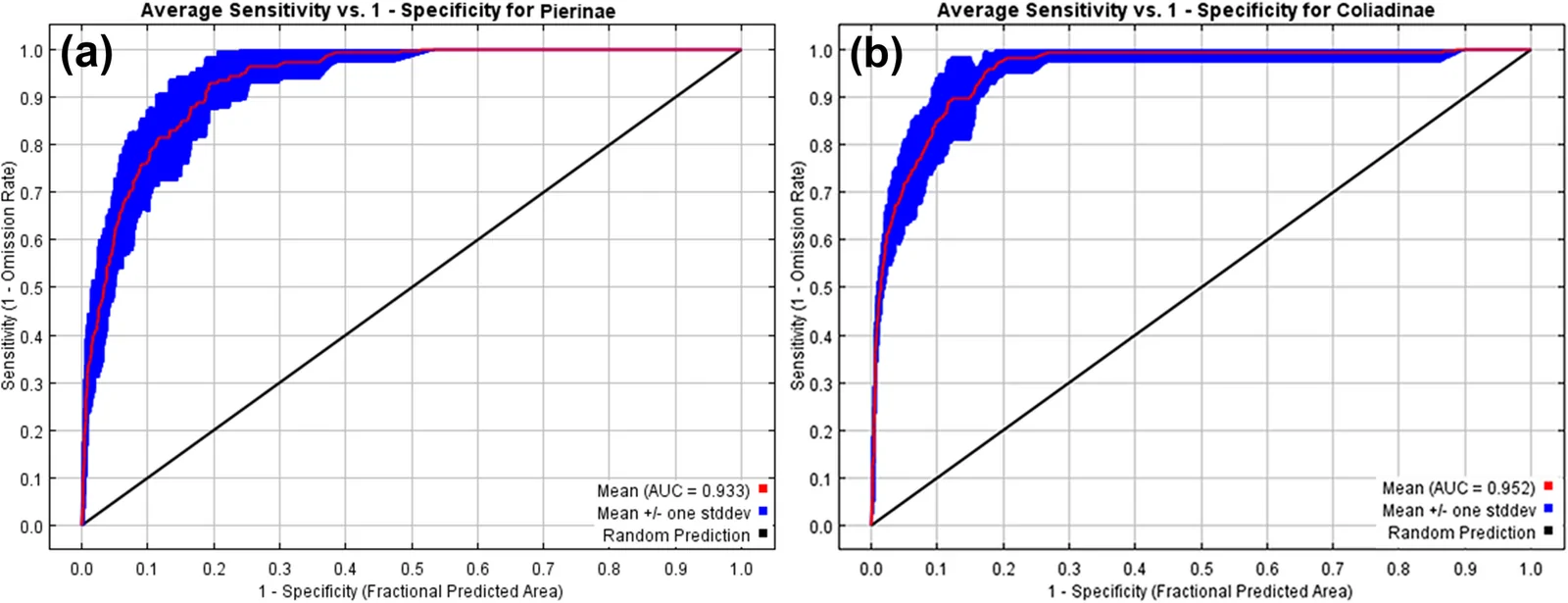
MaxEnt results for Pieridae across Taiwan Island: ROC curve for (**a**) Pierinae and (**b**) Coliadinae

The MaxEnt model showed relatively better performance, generally yielding AUC values of 0.933 for Pierinae (Fig. 3(a)) and 0.952 for Coliadinae (Fig. 3(b)). Furthermore, the AUC values were also specifically calculated for 23 species individually (Table S3). Similarly, the TSS values were generally calculated for both subfamilies and particularly for each individual species (Table S3). Generally, for both subfamilies and specifically for 23 individual species, the model was constructed utilizing six principal environmental parameters (Table S3). Fig. S4 shows the importance of six influential environmental factors for both subfamilies in the MaxEnt model. Elevation and Bio_5 are responsible for simulating the suitable habitats of Pierinae (80.6%) and Coliadinae (53.7%), respectively. The jackknife test reveals that for Pierinae (Fig. S12) and Coliadinae (Fig. S13), elevation and Bio_5 exhibited the largest increase when utilized independently and the most significant loss when omitted, respectively. For both subfamilies, response curves were generated for the top six important environmental factors (Figs. S14–S15). The response curves showed high distribution chances of Pierinae and Coliadinae when elevation is around zero and Bio_5 is from 32 to 34 °C, respectively.

## Simulation under historical periods

**Fig. 4:**
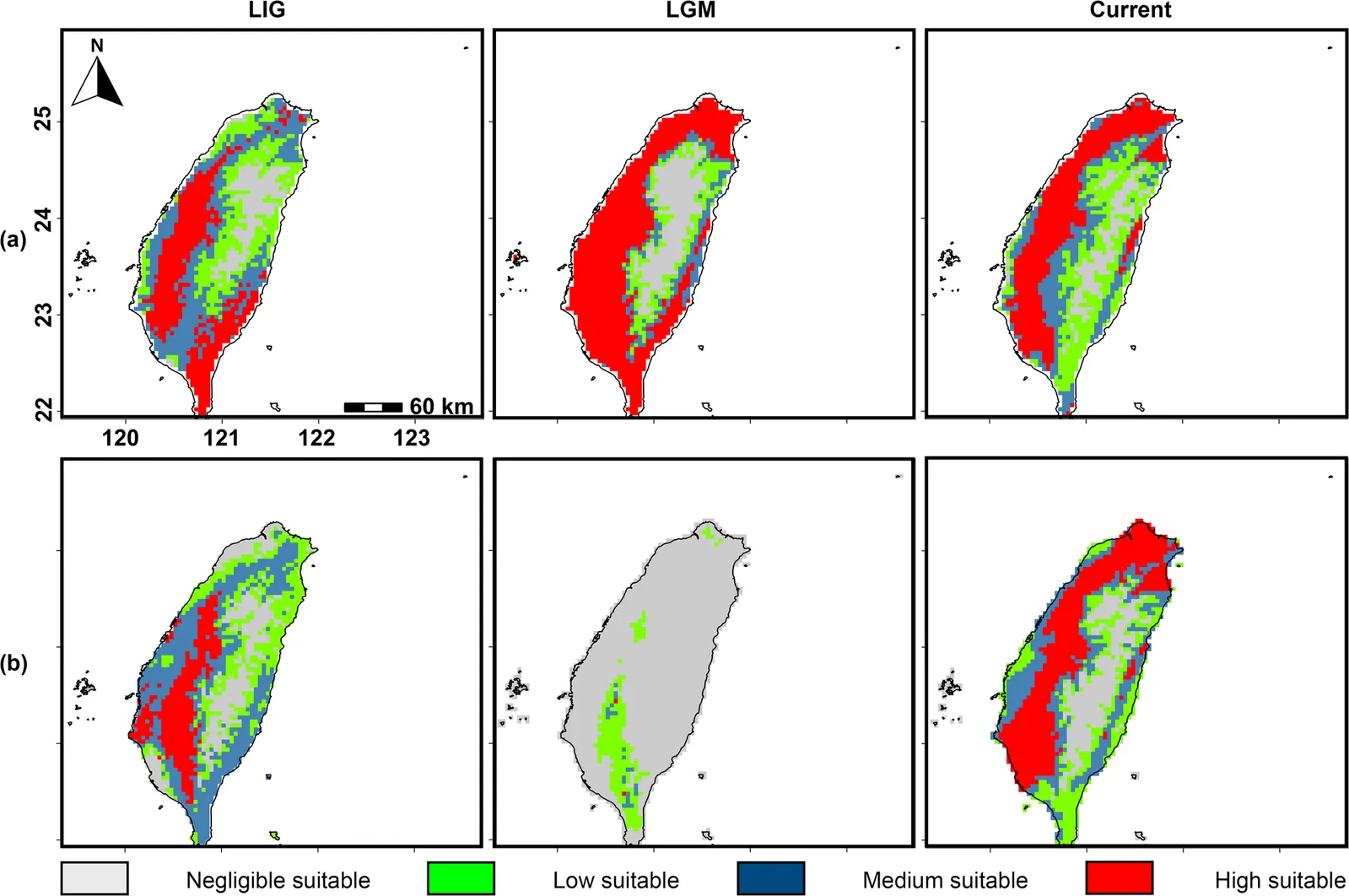
Historical (LIG, LGM, and Current) suitable areas for (**a**) Pierinae and (**b**) Coliadinae across Taiwan Island

Habitat suitability maps for Pierinae (Fig. 4(a)) and Coliadinae (Fig. 4(b)) were generated utilizing historical climate data. The prediction of suitable areas for Pierinae indicated that during the LIG period, the highly suitable areas were predominantly located in the southeastern (Taitung and Pingtung Counties) and western (Tainan, Chiayi, Yunlin, Changhua, Taichung, and Miaoli Counties) parts of Taiwan. From LIG to LGM, a decline in temperature resulted in an increase in areas highly suitable for Pierinae, expanding from 10,182.32 to 18,907.09 km^2^ (Table S6), particularly towards the southern (Kaohsiung County), central (western part of Nantou County), and northern (Hsinchu County, Taoyuan City, and Taipei City) parts of Taiwan.

Throughout the shift, most regions classified as medium- and low-suitable were transformed into high-suitable regions. From the LGM to Current period, the rise in temperature has resulted in a reduction of highly suitable areas from 18,907.09 to 12,308.41 km^2^ (Table S6), indicating a decline of 34.91%. During the transition, the zones deemed highly suitable in the southern and westernmost regions were transformed into low- and medium-suitable zones.

The prediction of suitable areas for Coliadinae showed that during the LIG period, the highly suitable zones were mainly located in Kaohsiung County, Tainan County, Chiayi County, Nantou County, and the northern part of Pingtung County. From LIG to LGM, a decrease in temperatures led to a near-total contraction of low-, medium-, and high-suitable regions for Coliadinae (Fig. 4(b); Table S6). From the LGM to Current period, the elevation in temperatures has led to a substantial expansion of highly suitable areas from 39.43 to 13,540.28 km^2^ (Table S6), signifying a 342-time increase. Throughout the transition, zones once deemed negligibly suitable in the western, eastern, and northern regions evolved into low-, medium-, and high-suitable regions.

To gain a more comprehensive understanding of each species individually, habitat suitability maps were created and area calculations were conducted. During the shift from LIG (Fig. S16) to LGM (Fig. S17), the highly suitable areas for 11 Pierinae species (*Appias albina*, *A. indra*, *A. libythea*, *Cepora nerissa*, *Delias hyparete*, *Hebomoia glaucippe*, *Ixias pyrene*, *Leptosia nina*, *Pieris rapae*, *Prioneris thestylis*, and *Talbotia naganum*) decreased, whereas those for 5 species (*Aporia agathon*, *Appias lyncida*, *Cepora nadina*, *Delias pasithoe*, and *Pieris canidia*) increased (Table S6). During the transition from the LGM (Fig. S17) to Current (Figs. S18–S19) period, the highly suitable areas for 12 Pierinae species (*A. albina*, *A. indra*, *A. libythea*, *C. nadina*, *C. nerissa*, *D. hyparete*, *H. glaucippe*, *I. pyrene*, *L. nina*, *P. canidia*, *P. rapae*, and *T. naganum*) increased, while those for 4 species (*A. agathon*, *A. lyncida*, *D. pasithoe*, and *P. thestylis*) decreased (Table S6). During the shift from LIG (Fig. S20) to LGM (Fig. S21), highly suitable habitats for 6 Coliadinae species (*Catopsilia pomona*, *Catopsilia pyranthe*, *Eurema andersoni*, *Eurema blanda*, *Eurema mandarina*, and *Gonepteryx amintha*) declined, while those for *Eurema hecabe* increased (Table S6). In contrast, from the LGM (Fig. S21) to Current (Fig. S22) period, the highly suitable areas for the above-mentioned 6 Coliadinae species increased, while those for *E. hecabe* decreased (Table S6).

## Simulation under future climate change scenarios

**Fig. 5:**
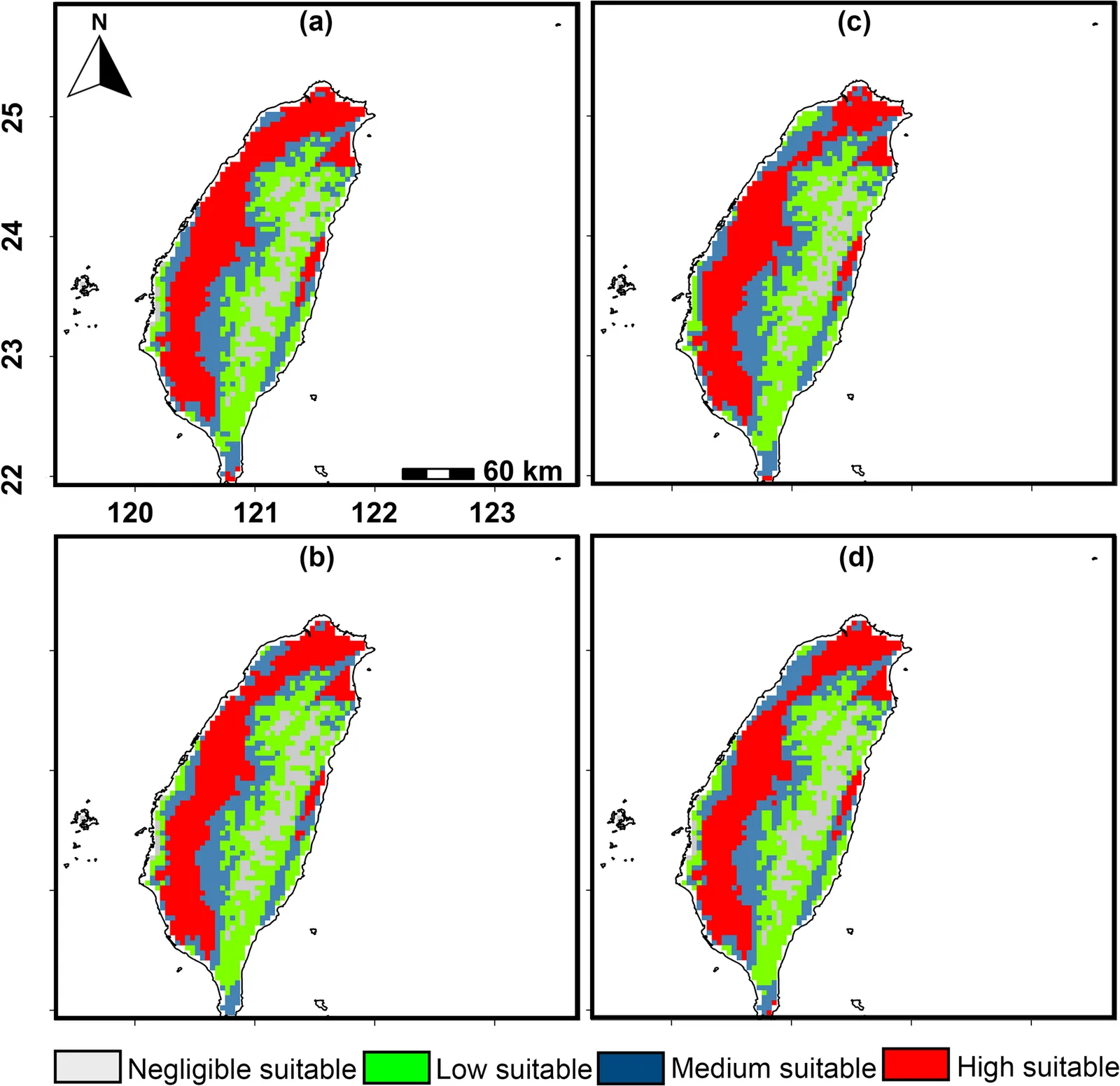
Future suitable areas of Pierinae on Taiwan Island in the 2030s and 2090s using the ACCESS-CM2 model: (**a**) 2030s–SSP1–2.6; (**b**) 2030s–SSP5–8.5; (**c**) 2090s–SSP1–2.6; and (**d**) 2090s–SSP5–8.5

The areas deemed highly suitable for Pierinae and Coliadinae are expected to reduce and remain confined at the same elevations in the future relative to their projected highly suitable regions under Current climatic conditions. Figure 5(a–b) illustrates the suitable regions for Pierinae under SSP1–2.6 scenarios. The areas identified as highly suitable in the Current period are projected to decline to 12,111.98 km^2^ by the 2030s (Fig. 5(a); Table S6) and 11,469.39 km^2^ by the 2090s (Fig. 5(b); Table S6). Similarly, Fig. 5(c–d) illustrates the suitable regions for Pierinae under SSP5–8.5 scenarios. The areas deemed highly suitable in the Current period are projected to decline to 11,280.15 km^2^ by the 2030s (Fig. 5(c); Table S6) and 11,081.10 km² by the 2090s (Fig. 5(d); Table S6).

**Fig. 6:**
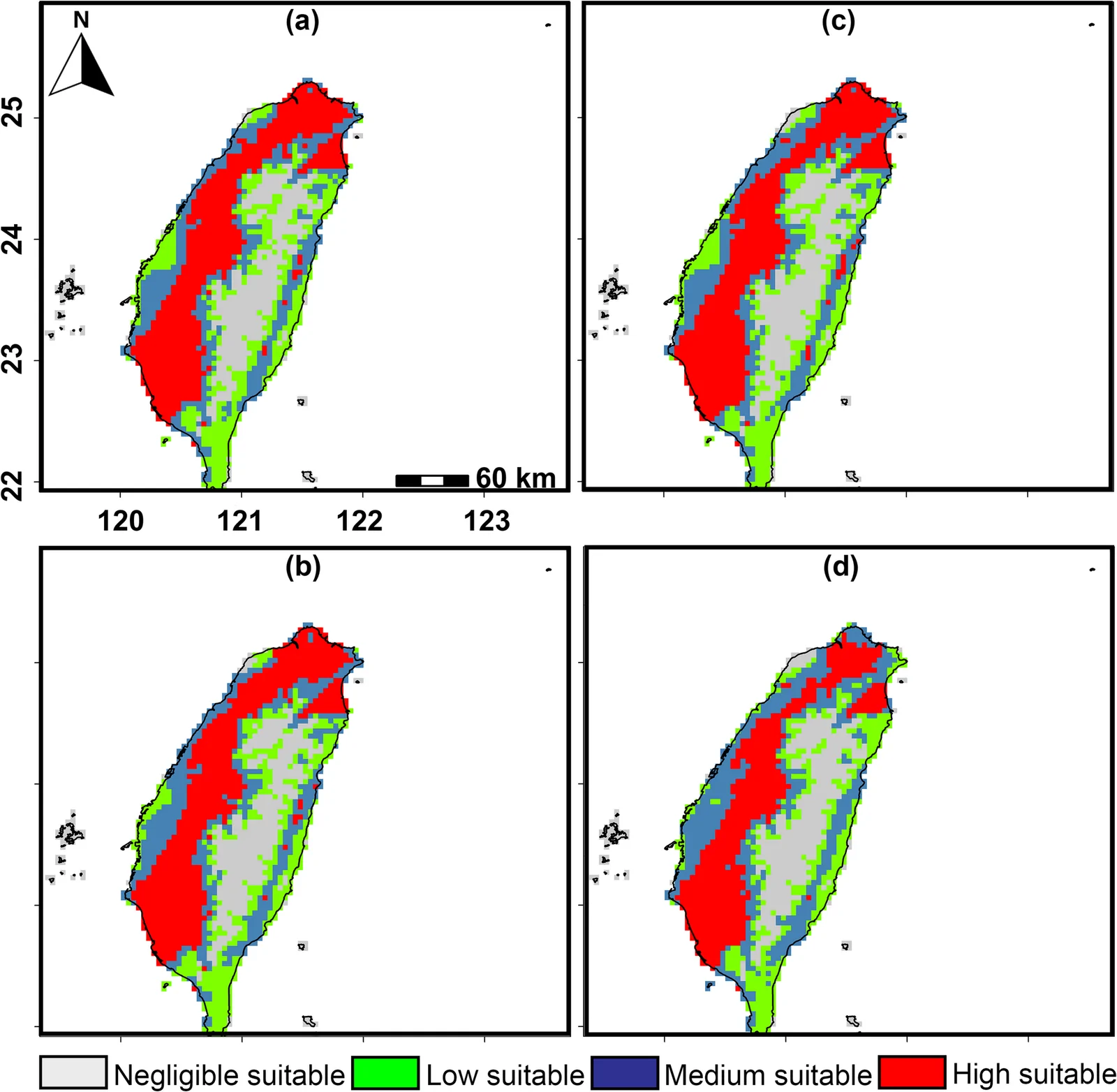
Future suitable area of Coliadinae on Taiwan Island in the 2030s and 2090s using the ACCESS-CM2 model. (**a**) 2030s–SSP1–2.6; (**b**) 2030s–SSP5–8.5; (**c**) 2090s–SSP1–2.6; and (**d**) 2090s–SSP5–8.5

Figure 6 illustrate the suitable areas for Coliadinae under SSP1–2.6 and SSP5–8.5 scenarios. Under the SSP1–2.6 scenarios, the identified highly suitable habitats during the Current period are predicted to decrease to 13,446.98 km^2^ (Fig. 6(a); Table S6] and 13,328.56 km^2^ (Fig. 6(c); Table S6) by the end of the 2030s and 2090s, respectively. Similarly, Fig. 6 illustrate the suitable regions for Coliadinae under SSP5–8.5 scenarios. The highly suitable habitats are predicted to decrease to 13,137.52 km² (Fig. 6(b); Table S6) and 11,266.39 km² (Fig. 6(d); Table S6) by the end of the 2030s and 2090s, respectively.

Habitat suitability maps were generated, and area calculations were performed to strengthen the knowledge about each species in the context of climate change scenarios. Generally, it is predicted that most species will experience a loss of suitable habitats by the 2090s and will remain confined to the same elevations. Under SSP1–2.6 scenarios, compared to the Current period, the highly suitable areas for 11 Pierinae species are projected to decline by the 2090s, while those for 5 species are anticipated to rise (Figs. S18–S19; Table S6). Under SSP5–8.5 scenarios, the highly suitable regions for all Pierinae species are projected to decline by the 2090s (Figs. S18–S19; Table S6).

Under SSP1–2.6 scenarios, compared to the Current period, the highly suitable habitats for 6 Coliadinae species are expected to decrease by the 2090s, while those for *C. pyranthe* are predicted to increase (Fig. S22; Table S6). Under SSP5–8.5 scenarios, the highly suitable regions for 5 Coliadinae species are projected to decrease by the 2090s, whereas those for *E. mandarina* and *G. amintha* are predicted to increase (Fig. S22; Table S6).

## Future elevation shift

**Fig. 7:**
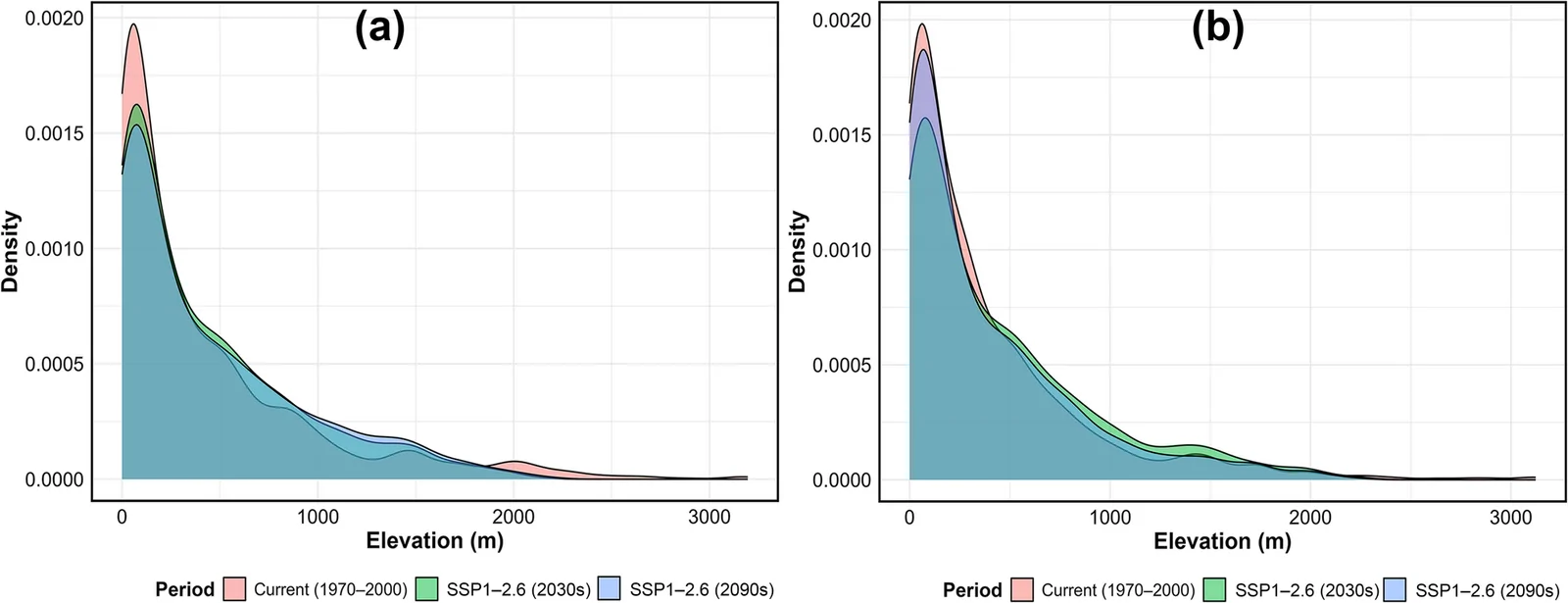
Assessment of the variations in elevation of suitable (**a**) Pierinae and (**b**) Coliadinae habitats across Taiwan Island under the Current period and projected climate change scenarios

The elevation-based comparison under the Current period and future climate change scenarios (SSP1–2.6) indicated no substantial change in the elevation of Pierinae and Coliadinae. The average elevation of occurrences during the Current period was 430 m for Pierinae and 384 m for Coliadinae. However, the estimates of future climate change scenarios suggested comparable average altitudes of 434 m in the 2030s and 445 m in the 2090s for Pierinae (Fig. 7(a)), as well as 385 m in the 2030s and 386 m in the 2090s for Coliadinae (Fig. 7(b)). Welch’s two-sample t-tests indicated no substantial variations between the Current and future elevations for both Pierinae (2030s: *t* = − 0.77, *p* = 0.47; 2090s: *t* = − 0.76, *p* = 0.45) and Coliadinae (2030s: *t* = − 0.81, *p* = 0.95; 2090s: *t* = − 0.8, *p* = 0.94), implying that appropriate areas are not migrating upstream.

## Discussion

Global warming, coupled with urbanization, rising land surface temperatures, habitat destruction, ecological instability, the invasion of non-native species, and decreasing available space, are key determinants of changes in biodiversity distribution patterns, species loss, and habitat reduction [5, 7, 54]. This phenomenon is especially evident in climate-sensitive and endemic species, which exhibit elevated sensitivity to even slight changes in climate and habitat composition [1, 2, 7]. Understanding these processes is essential for interpreting insect responses, particularly in butterflies, whose distributions depend on regional climate and the availability of food and host plants [1, 2, 4, 7, 55]. This study points out the necessity of simultaneously considering both biological and climatic constraints when examining potential distribution patterns [1, 2, 56]. Against this backdrop, temperature and precipitation variability, elevational diversity, plant composition, and anthropogenic activities emerge as important drivers of butterfly spatial distribution and diversity. These variables influence physiological tolerance, host-plant presence, and habitat appropriateness throughout different landscapes [1, 2, 4, 7, 57]. In this research, both Pierinae (mean elevation: 429 m) and Coliadinae (mean elevation: 333 m) were mainly limited to lowland areas across Taiwan Island, where steep topography and lower temperatures at greater elevations likely impose physiological constraints, thereby limiting appropriate regions. In contrast, across mainland China, both Pierinae (mean elevation: 819 m) and Coliadinae (mean elevation: 897 m) occupied a wider elevational range, extending into higher elevations before being limited by decreased precipitation and temperatures at mountain tops. This difference highlights the greater environmental and topographic diversity of mainland ecosystems, which provides a vast ecological space and better buffering capacity compared to island ecosystems [1, 2, 7, 58].

The MaxEnt model results for Taiwan reveal different ecological sensitivities between Pierinae and Coliadinae, with elevation (particularly lowland areas) being the main factor for Pierinae and temperature (Bio5) playing a key role for Coliadinae, underlining subfamily-specific thermal and habitat limits. Our results are consistent with earlier studies conducted in China and the French Alps [1, 2, 7, 57]. Furthermore, the positive associations of Pierinae and Coliadinae with anthropogenic activities in higher-elevation regions of Taiwan likely reflect limited space and increased anthropogenic activities at lower elevations. The positive association between Pierinae and plant cover in lowland areas highlights the importance of host plants and food resources, particularly in areas affected by anthropogenic activities. In contrast, in mainland China, both subfamilies illustrated negative relationships with anthropogenic activities and vegetation cover at greater elevations, suggesting greater susceptibility to human disturbance and indicating a tendency to persist in less disturbed environments with relatively lower vegetation density. These results are in line with earlier studies demonstrating that temperature, precipitation, topographic diversity, anthropogenic activities, and vegetation density jointly shape the distribution patterns of butterflies [1, 2, 7, 59, 60].

Studying butterfly dispersal throughout different climatic eras shows significant distributional patterns, giving valuable insights into historical biogeography, the recognition of refugia, prediction of species vulnerability to ecological variation, and the delineation of priority conservation regions. Importantly, integrating distributional changes from the LIG to future projections enables a comprehensive understanding of species’ long-term responses to climatic fluctuations, thereby improving the reliability of future predictions under ongoing global change [1, 15]. From the LIG to LGM, this study demonstrated a general increase in highly suitable regions for Pierinae, while Coliadinae experienced a general decrease in highly appropriate habitats. These patterns are in line with prior research on butterflies and other insects, which demonstrates that closely related taxa can respond differently to past climatic changes due to species-specific ecological traits, for example, thermal tolerance, host-plant specialization, and dispersal capacity, resulting in divergent patterns of range expansion and contraction during glacial-interglacial cycles [1, 61, 62]. In mainland China, large land area and environmental heterogeneity facilitated habitat expansion, whereas in island ecosystems, limited area, isolation, and restricted ecological refugia constrained such expansion. Furthermore, decreased dispersal pathways and interspecific variation in physiological tolerance may constrain the capability of some Pierid species to adjust their habitats in response to climate change, a constraint that is comparatively weaker in mainland systems [1, 63]. Per-species analyses showed that 5 Pierinae species and one Coliadinae species (*E. hecabe*) exhibited increases in highly suitable habitats, while the remaining species (11 Pierinae and 6 Coliadinae) showed declines. While both subfamilies generally prefer warmer environments, the rise in Pierinae-suitable habitats during the LGM may be attributed to host-plant availability, environmental resilience, evolutionary history, and microhabitat availability [1, 64, 65].

Subsequent to the ice age, worldwide temperatures increased, leading to a decrease in highly suitable habitats for Pierinae. At the species level, 12 Pierinae species displayed a rise in highly appropriate regions, while 4 species decreased. In contrast, Coliadinae showed a sharp rise in suitable regions, extending throughout Taiwan Island. In total, 6 Coliadinae species showed a sharp rise in highly suitable regions, whereas one species (*E. hecabe*) decreased. These contrasting responses likely arise from differences in ecological traits, specifically temperature and humidity tolerance limits, host-plant specificity, and dispersal capability, which affect their potential to exploit newly appropriate habitats under post-glacial warming. These changes also reflect the inherent constraints of island ecosystems, including habitat fragmentation, which increase species dependence on specific habitat types and localized elevational gradients [1, 61, 62, 64, 65].

The IPCC-AR4 projections estimated that, by 2100, temperature and precipitation could increase by 1.6–5 ℃ and 1.5–20%, respectively [66]. Over the past century, mean surface air temperature has increased by approximately 0.03–0.12 °C per decade [67]. Therefore, around 24% of terrestrial species are likely to be at risk of extinction in the near future [68]. Research indicates that a 2 − 3 ℃ increase in average global temperature could lead to the extinction of 20 − 30% of animal and plant species [69]. Insects, particularly butterflies, exhibit heightened sensitivity to temperature variations owing to their restricted thermoregulatory capacity, small population sizes, and narrow habitat ranges [1, 2, 7, 70]. In this study, both Pierinae and Coliadinae showed a general shrinkage in highly appropriate areas under anticipated climate change scenarios. Under SSP1–2.6 and SSP5–8.5 scenarios, by the 2090s, per-species analysis indicates that most species are expected to face habitat shrinkage. Such reductions may increase the risk of local eradication, as dispersal toward newly suitable regions is constrained by steep elevation, adverse environmental conditions, and decreased availability of host plants and food resources. These findings are consistent with previous research, which reports that global warming may cause the loss of 64% of butterfly species in tropical mountains by 2070 [55], and studies in mainland China and Taiwan have also projected future declines in appropriate regions for the majority of butterfly species [1, 2, 7, 9].

Despite these predicted decrease, uphill movement is a well-known response of butterflies to global warming, particularly in continental systems [1, 2, 7]. In contrast, our findings suggest that, throughout Taiwan Island, Pierinae and Coliadinae are expected to remain within broadly similar elevational zones under future climate scenarios, rather than showing pronounced uphill shifts. The limited land area, steep topography, sharp decrease in temperatures with rising elevation, and restricted habitat availability on islands create substantial barriers to species movement, thereby limiting range expansion into newly suitable areas [5, 71–73]. Unlike mainland ecosystems, where elevational shifts can buffer species against warming [1, 2, 7], island ecosystems, such as Taiwan, offer fewer climatic refugia and more restricted dispersal routes, which increase the impacts of climate change. Furthermore, the strong dependence of butterflies on specific larval host plants and nectar resources—whose distributions may also be limited by island topography and climate—can further limit their ability to shift elevationally, thereby contributing to the observed stability in distribution patterns [74, 75]. This trend indicates that climate-driven habitat change is multifaceted, influenced not only by temperature but also by the speed of regional environmental change, precipitation patterns, and local habitat structure. Furthermore, microhabitat diversity across the study area may provide localized refugia, permitting species to keep their present distributions rather than moving upslope [64, 65, 71, 76]. This highlights the importance of conserving microhabitat heterogeneity to support butterfly persistence under future climate change.

This study predicts that global warming will lead to a decrease in Pieridae diversity across Taiwan Island and that their ability to track climate change through uphill movement is limited, leaving them severely exposed to high anthropogenic activities in lowland areas. Accordingly, conservation strategies should emphasize the preservation and restoration of suitable regions at lower elevations, with particular focus on protecting and improving semi-natural regions, transit corridors, and host plant resources. Effective conservation efforts should focus on reducing habitat fragmentation, restoring degraded patches, decreasing human-caused pressures, and maintaining microhabitats that provide local climatic buffering. Given the restricted dispersal capability of island species, island-specific conservation strategies should also implement ex-situ measures, specifically for species at high risk of local extinction. Butterfly protection initiatives worldwide highlight the necessity of developing and implementing management methods that address the integrated dangers of global warming and habitat loss, especially in highly anthropogenic areas [1, 2, 7, 55, 77]. Targeted habitat restoration can decrease biodiversity loss and, in some cases, facilitate recovery of threatened butterfly populations, indicating that appropriate management interventions can significantly influence outcomes under multiple environmental pressures [78]. Therefore, identifying and predicting suitable habitats for butterflies is important for effective conservation planning [1, 2].

Based on these conservation implications, this study investigated both past and future distribution patterns of Pierinae and Coliadinae to offer a detailed framework for their protection. The suitable areas identified for both subfamilies can assist policymakers and conservation institutions in determining crucial conservation regions, particularly in highly developed landscapes [1, 7, 43]. Such approaches may guide future conservation efforts to protect butterfly populations where uphill shifts in response to climate change are restricted and anthropogenic activities are more intense at lower elevations. In this research, we used two types of environmental datasets (19 bioclimatic and 2 topographic variables) to reconstruct historical distributions and predict future scenarios for both subfamilies. Moreover, we assessed the influence of 25 environmental variables on the distribution of both subfamilies across Taiwan Island and mainland China along elevational gradients. However, our study has several limitations that should be addressed in future studies. Specifically, we did not incorporate biotic interactions (e.g., competition, predation, food availability, and host-plant accessibility), although prior research suggests that excluding these factors may lead to underestimation of habitat appropriateness [4, 79]. Furthermore, edaphic variables were not included, despite their recognized significance in butterfly ecology [80]. Our findings indicate a general decrease in suitable habitats under future global warming scenarios; therefore, continuous monitoring of butterfly distributions, along with the incorporation of both environmental and non-environmental variables, will be necessary for developing significant conservation measures.

## Conclusion

The current research highlighted the significant ecological differentiation between island and mainland butterfly populations, shaped by discrete environmental gradients, human pressure, and elevational patterns. On Taiwan Island, the average elevation of both subfamilies is comparatively lower due to the sharp decline in temperatures with a sharp increase in elevation. Conversely, in mainland China, both subfamilies are distributed at significantly elevated average elevations owing to reduced temperature and precipitation at higher elevations and diverse topography. On Taiwan Island, both subfamilies showed positive associations with human disturbances at greater elevations, while Pierinae preferred enhanced vegetation cover at lower elevations. In mainland China, both subfamilies display negative relationships with human-induced processes and plant cover at greater elevations. According to the MaxEnt model, on Taiwan Island, elevation and Bio_5 are the most important determinants influencing Pierinae and Coliadinae distribution, respectively. From LIG to LGM and LGM to Current periods, Pierinae exhibited an initial increase in highly suitable areas followed by a decrease, while Coliadinae showed an initial decline followed by a rise. Future projection analysis indicated that the highly suitable habitats for Pierinae and Coliadinae are anticipated to decrease significantly, without clear signs of shifting towards higher elevations. Most of the species are expected to contract their suitable habitats, leading to a decline in Pieridae species diversity.

This study suggests that conservation policies must be region-specific, targeting elevational linkage and lowland habitat conservation in insular systems while maintaining broad-scale climatic refugia across mainland ecosystems to ensure extended survival of butterfly populations under rising global warming. Subsequent studies should focus on biotic relationships, edaphic variables, and continuous monitoring of Pieridae distribution trends to enhance their prediction accuracy and conservation.

## Supplementary Information

Below is the link to the electronic supplementary material.

Supplementary Material 1

Supplementary Material 2

## Data Availability

The data presented in this study are available on request from the corresponding author.
