# Supplementary material for: Elevational constraints and climatic buffering: comparison of Pierinae and Coliadinae across Taiwan Island and mainland China
Source: Front Zool. 2026 Jun 22;23:31. doi: 10.1186/s12983-026-00621-6 (PMC13543383; doi:10.1186/s12983-026-00621-6)
Supplement: Supplementary file 1 [file 12983_2026_621_MOESM1_ESM.zip › Supplementary Tables/Legend of Tables.docx]

**Table S1**. Information of 26 environmental variables.

**Table S2**. Percent (%) contributions of 19 bioclimatic and two topographic factors used in the MaxEnt model for Pierinae and Coliadinae across Taiwan Island.

**Table S3**. Summary of the MaxEnt model settings and performance metrics for Pierinae and Coliadinae, together with their respective individual species.

**Table S4**. Linear mixed-effects model (LLM) results of 25 predictors against the elevation gradient for Pierinae and Coliadinae distribution on Taiwan Island.

**Table S5**. Linear mixed-effects model (LLM) results of 25 predictors against the elevation gradient for Pierinae and Coliadinae distribution in mainland China.

**Table S6**. Changes in the area (km²) of suitable habitats for Pieridae (Pierinae and Coliadinae) across Taiwan Island in different periods (LIG, LGM, Current, and global warming scenarios). Blue color indicates an increase in highly suitable areas, and red color denotes a decrease in highly suitable areas compared to the Current period.
