# Supplementary material for: Elevational constraints and climatic buffering: comparison of Pierinae and Coliadinae across Taiwan Island and mainland China
Source: Front Zool. 2026 Jun 22;23:31. doi: 10.1186/s12983-026-00621-6 (PMC13543383; doi:10.1186/s12983-026-00621-6)
Supplement: Supplementary file 2 [file 12983_2026_621_MOESM2_ESM.zip › Supplementary Figures/Supplementary Figures.docx]

**Fig. S1** Pieridae (Pierinae and Coliadinae) species diversity across Taiwan Island.

**Fig. S2** Correlation analysis of climatic variables with Pierinae across Taiwan Island. Note that the right portion represents the correlation value *r*, varying from −1 to 1. The positive numbers show positive associations, negative numbers show negative associations, and 0 shows no correlation. The higher the value of |*r*|, the more significant the association. The upper right portion is a graphical transformation of correlation values. Positive associations are displayed in blue and negative associations in red color. Color intensity and size of the circle are proportional to the correlation coefficients (refer to the legend on the right).

**Fig. S3** Correlation analysis of climatic variables with Coliadinae across Taiwan Island. Note that the right portion represents the correlation value *r*, varying from −1 to 1. The positive numbers show positive associations, negative numbers show negative associations, and 0 shows no correlation. The higher the value of |*r*|, the more significant the association. The upper right portion is a graphical transformation of correlation values. Positive associations are displayed in blue and negative associations in red color. Color intensity and size of the circle are proportional to the correlation coefficients (refer to the legend on the right).

**Fig. S4** Percent contribution of environmental variables used in the final MaxEnt models for Pierinae and Coliadinae. Higher values indicate the greater importance of variables in predicting habitat suitability. Orange color shows Pierinae, and sky blue color represents Coliadinae.

 **Fig. S5** Species accumulation curves for Pieridae on Taiwan Island and mainland China based on bootstrap mean values: (**a**) Pierinae on Taiwan Island; (**b**) Coliadinae on Taiwan Island; (**c**) Pierinae in mainland China (**d**); Coliadinae in mainland China.

 **Fig. S6** Correlation analysis between elevation and environmental variables for subfamily (**a**) Pierinae and (**b**) Coliadinae across Taiwan Island. Bars illustrate the orientation of the correlation (Pearson’s *r*), with green bars denoting positive correlations (factors that increase with elevation) and red bars signifying negative correlations (factors that drop with elevation). Color intensities are proportional to the correlation coefficients (refer to the legend on the right).

**Fig. S7** Correlation analysis between elevation and environmental variables for subfamily (**a**) Pierinae and (**b**) Coliadinae across mainland China. Bars illustrate the orientation of the correlation (Pearson’s *r*), with green bars denoting positive correlations (factors that increase with elevation) and red bars signifying negative correlations (factors that drop with elevation). Color intensities are proportional to the correlation coefficients (refer to the legend on the right).

**Fig. S8** Comparison of essential ecological factors between low- and high-elevation locations for the subfamily Pierinae throughout Taiwan Island. Each graph illustrates the distribution of an environmental factor among low-elevation (green) and high-elevation (red) groups through integrated violin and boxplots, with the violin outlines showing data distribution and the inside boxplots displaying the median and interquartile ranges. Blue diamonds represent mean values. The significance levels from the two-sample t-tests are displayed on the top left of each comparison, indicating *p*-values with significance codes (< 0.001).

**Fig. S9** Comparison of essential ecological factors between low- and high-elevation locations for the subfamily Coliadinae throughout Taiwan Island. Each graph illustrates the distribution of an environmental factor among low-elevation (green) and high-elevation (red) groups through integrated violin and boxplots, with the violin outlines showing data distribution and the inside boxplots displaying the median and interquartile ranges. Blue diamonds represent mean values. The significance levels from the two-sample t-tests are displayed on the top left of each comparison, indicating *p*-values with significance codes (< 0.001).

**Fig. S10** Comparison of essential ecological factors between low- and high-elevation locations for the subfamily Pierinae throughout mainland China. Each graph illustrates the distribution of an environmental factor among low-elevation (green) and high-elevation (red) groups through integrated violin and boxplots, with the violin outlines showing data distribution and the inside boxplots displaying the median and interquartile ranges. Blue diamonds represent mean values. The significance levels from the two-sample t-tests are displayed on the top left of each comparison, indicating *p*-values with significance codes (< 0.001).

**Fig. S11** Comparison of essential ecological factors between low- and high-elevation locations for the subfamily Coliadinae throughout mainland China. Each graph illustrates the distribution of an environmental factor among low-elevation (green) and high-elevation (red) groups through integrated violin and boxplots, with the violin outlines showing data distribution and the inside boxplots displaying the median and interquartile ranges. Blue diamonds represent mean values. The significance levels from the two-sample t-tests are displayed on the top left of each comparison, indicating *p*-values with significance codes (< 0.001).

**Fig. S12** Pierinae Jackknife test result for the most influential six variables: (**a**) regularized training gain; (**b**) AUC values; and (**c**) test gain.

**Fig. S13** Coliadinae Jackknife test result for the most influential six variables: (**a**) regularized training gain; (**b**) AUC values; and (**c**) test gain.

**Fig. S14** Response curves for the six major environmental variables of Pierinae. Red lines show the mean response, and blue margins are ± SD calculated over 10 replicates.

**Fig. S15** Response curves for the six major environmental variables of Coliadinae. Red lines show the mean response, and blue margins are ± SD calculated over 10 replicates.

**Fig. S16** Suitable areas for Pierinae species during the LIG period across Taiwan Island: (**a**) *Aporia agathon*, (**b**) *Appias albina*, (**c**) *A. indra*, (**d**) *A. libythea*, (**e**) *A. lyncida*, (**f**) *Cepora nadina*, (**g**) *C. nerissa*, (**h**) *Delias hyparete*, (**i**) *D. pasithoe*, (**j**) *Hebomoia glaucippe*, (**k**) *Ixias pyrene*, (**l**) *Leptosia nina*, (**m**) *Pieris canidia*, (**n**) *P. rapae*, (**o**) *Prioneris thestylis*, and (**p**) *Talbotia naganum*.

**Fig. S17** Suitable regions for Pierinae species in the LGM period throughout Taiwan Island: (**a**) *Aporia agathon*, (**b**) *Appias albina*, (**c**) *A. indra*, (**d**) *A. libythea*, (**e**) *A. lyncida*, (**f**) *Cepora nadina*, (**g**) *C. nerissa*, (**h**) *Delias hyparete*, (**i**) *D. pasithoe*, (**j**) *Hebomoia glaucippe*, (**k**) *Ixias pyrene*, (**l**) *Leptosia nina*, (**m**) *Pieris canidia*, (**n**) *P. rapae*, (**o**) *Prioneris thestylis*, and (**p**) *Talbotia naganum*.

**Fig. S18** Suitable habitats for Pierinae species in the Current period (1970–2000) and under future global warming scenarios (ACCESS-CM2 model) across Taiwan Island. The suitable habitats of each species in the Current period are given on the left side, while their corresponding suitable habitats under the future global warming scenarios are given on the right side. The names of species are: (**a**) *Aporia agathon*, (**b**) *Appias albina*, (**c**) *A.* *indra*, (**d**) *A. libythea*, (**e**) *A. lyncida*, (**f**) *Cepora nadina*, (**g**) *C. nerissa*, and (**h**) *Talbotia naganum*.

**Fig. S19** Suitable regions for Pierinae species in the Current period (1970–2000) and under future global warming scenarios (ACCESS-CM2 model) throughout Taiwan Island. The suitable habitats of each species in the Current period are given on the left side, while their corresponding suitable habitats under the future global warming scenarios are given on the right side. The names of species are: (**a**) *Delias hyparete*, (**b**) *D. pasithoe*, (**c**) *Hebomoia glaucippe*, (**d**) *Ixias pyrene*, (**e**) *Leptosia nina*, (**f**) *P. canidia*, (**g**) *P. rapae*, and (**h**) *Prioneris thestylis*.

**Fig. S20** Suitable habitats for Coliadinae species in the LIG period across Taiwan Island: (**a**) *Catopsilia pomona*, (**b**) *C. pyranthe*, (**c**) *Eurema andersoni*, (**d**) *E. blanda*, (**e**) *E. hecabe*, (**f**) *E. mandarina*, (**g**) *Gonepteryx amintha*.

**Fig. S21** Suitable areas for Coliadinae species in the LGM era throughout Taiwan Island: (**a**) *Catopsilia pomona*, (**b**) *C. pyranthe*, (**c**) *Eurema andersoni*, (**d**) *E. blanda*, (**e**) *E. hecabe*, (**f**) *E. mandarina*, (**g**) *Gonepteryx amintha*.

**Fig. S22** Suitable habitats for Coliadinae species in the Current period (1970–2000) and under future global warming scenarios (ACCESS-CM2 model) across Taiwan Island. The suitable habitats of each species in the Current period are given on the left side, while their corresponding suitable habitats under the future global warming scenarios are given on the right side. The names of species are: (**a**) *Catopsilia pomona*, (**b**) *C. pyranthe*, (**c**) *Eurema andersoni*, (**d**) *E. blanda*, (**e**) *E. hecabe*, (**f**) *E. mandarina*, and (**g**) *Gonepteryx amintha*.
